# Influence of Post-Consumer Waste Thermoplastic Elastomers Obtained from Used Car Floor Mats on Concrete Properties

**DOI:** 10.3390/ma16062231

**Published:** 2023-03-10

**Authors:** Alina Pietrzak, Malgorzata Ulewicz

**Affiliations:** Faculty of Civil Engineering, Czestochowa University of Technology, Dabrowskiego 69 Street, PL 42-201 Czestochowa, Poland

**Keywords:** concrete, compressive strength, frost resistance, microstructure, post-consumer waste, thermoplastic elastomer

## Abstract

In this paper, the influence of post-consumer thermoplastic elastomer (TPE) additive derived from used car floor mats on the physical and mechanical properties of concrete is presented. Waste elastomer (fractions 0–2 and 2–8 mm) was used as a substitute for sand or fine aggregate in the amount of 2.5, 5.0, 7.5, and 10.0% by weight of cement. For all series, the physical and mechanical properties of concrete (for example, compressive strength, flexural tensile strength, water absorption, density, and frost resistance), as well as its microstructure, were tested. It has been shown that post-consumer elastomer waste from used car floor mats in the amount of 2.5% of cement weight can replace sand and gravel aggregate in concrete without reducing their mechanical strength and without changing their microstructure. The compressive strength (after 28 days) of concretes in which the waste was introduced as a substitute for sand and aggregate was 57.0 and 57.2 MPa, respectively (the strength of the control sample was 57.0 MPa). The use of post-consumer waste in concrete allows for a reduction in the consumption of natural aggregate (the addition of 2.5% of waste material saves natural aggregate approximately 20 kg/m^3^), which reduces the cost of concrete production and also has a positive impact on the environment (i.e., it saves cost and space in landfills, where currently used car floor mat are deposited).

## 1. Introduction

Currently, there are a large number of polymeric waste materials on the market that have no practical use. This group includes used car floor mats marked with waste code: 16 01 19 (i.e., waste not included in other groups—used or unusable vehicles, waste from dismantling, inspection, and maintenance of vehicles—plastics) [1]. Considering that in 2020, in Poland alone, 479,900 end-of-life cars were dismantled, with each of them equipped with at least four car mats, and more than 1.92 million pieces of this waste ended up in landfills [2]. On the scale of the European Union or the world, this problem is particularly important, as there is currently no effective way to manage this type of waste. In Poland, in 2023, for depositing used floor car mats at a landfill, an environmental fee must be paid (the method of recycling this waste is unknown) in the amount of PLN 300.17 per 1 ton of waste (price list following the fee of the Minister of Climate and Environment—MP 2022, item 1009). The diverse chemical composition of the main polymer and the diverse composition of the modifying additives used to make car linings at different times make the segregation of this type of waste ineffective and chemical recycling impossible. Car floor mats are usually made of needle velour (polypropylene or polyamide) finished with rubber crumbs (e.g., styrene–butadiene, butadiene–acrylonitrile, polybutadiene, or polyolefin). Moreover, this material contains modifying additives, such as plasticizers, reinforcing fillings, flame retardant agents, UV stabilizers, mineral fillings, antioxidants, and coloring agents. The composition of the floor coverings varies depending on the year of production, the manufacturer, and the technology used. Therefore, currently, such chemically diverse waste is deposited in landfills and an effective management technology is sought for them.

Bearing in mind how important the problem for the environment is the management of used car floor coverings, in this work we attempted to determine the possibility of using this type of waste to produce concrete. The basis for this assumption were numerous review papers [3,4,5,6,7,8] on the use of polymer waste for concrete production and satisfactory results on the use of post-production waste of thermoplastic elastomers from the production of car floor mats, as presented in our previous paper [9]. It was shown that the addition of waste thermoplastic elastomer from the production process of car floor mats in the amount of 2.5% of cement mass to concrete as an aggregate substitute did not reduce the mechanical properties of concrete. There are many reports in the literature concerning the possibility of using other, various polymer wastes as an additive to concrete. According to the literature [10,11,12,13,14,15,16,17,18], polyterephthalate (PET) is the most extensively studied in terms of its use in concrete technology. Waste polyterephthalate is used in concrete mainly in two forms: as aggregate (coarse and fine) replacing natural aggregate and as polymer fiber. It was shown that the method of preparation and the shape of PET recyclate affect the parameters of concrete. Recyclate with a smooth, spherical surface has less impact on the workability of concrete than recyclate with a heterogeneous shape. The addition of PET recyclate increases [10,17] or decreases [12,14,15,16] the compressive strength of concrete. This strength depends to a large extent on the amount of added waste [13]. In addition, the flexural strength of concretes with the addition of waste PET may be higher [10,17] or lower [11,12,15,16] than the control concrete samples. The addition of PET recyclate to concrete reduces the modulus of elasticity, regardless of the tested consistency and the water–cement ratio. Most studies have shown an increase in water absorption with increasing PET waste inside concrete [18].

Moreover, post-production waste and post-consumer polystyrene waste were used for the production of concrete on a laboratory scale. This material was used both in the form of ball-shaped regranulate [19,20,21,22,23] and pellets [24,25,26,27,28,29,30,31] as an aggregate substitute in amounts from 10% to even 100%. The resulting composite was characterized by low density and better thermal parameters compared to ordinary concrete. The density of the modified concrete decreased with the increase in the amount of added polystyrene. The compressive strength of concrete depends, to a large extent, on the amount of waste added. The addition of polystyrene recyclate increases [20,21,22,23] or decreases [24,25,26,27,28,29,30,31] the compressive strength of concrete compared to control concrete samples. In addition, recyclate from waste polyethylene and polypropylene was used to produce concrete [32,33,34,35,36,37,38,39]. This material was used as a substitute for aggregate or as concrete reinforcement. Recycling mixtures of this raw material are characterized by varied quality and mechanical properties. Since the properties of fibers made of pure synthetic polymer differ significantly from fibers obtained from recycled material, fibers made of pure polypropylene or polyethylene were more often used in the study. Concretes containing 1% polypropylene fibers show higher compressive and splitting or bending strength than a series of controlled concretes. In addition, the addition of polypropylene recyclate increases the compressive strength [33,35,39] and flexural strength [35,39] of concrete concerning the control samples.

There are also several literature reports on the use of rubber in the production of concrete [40,41,42,43,44,45,46]. Concretes with the addition of rubber recyclate are usually characterized by lower values of mechanical parameters than concretes produced without the addition of waste [41,43,45,46,47], although there are also reports [40] that the addition of this material does not affect the mechanical properties of concrete. The decrease in the compressive strength after adding waste rubber, as shown in the works, is mainly related to the size of the introduced waste material. As shown in [42], the addition of recyclate (rubber from tire treads) in the amount of 5 and 10% with a grain size of 0.59 mm reduces the compressive strength of concrete by 61.5 and 88.5%, respectively. However, after adding waste with a grain size of 0.29 mm, the compressive strength was 70.9 and 97.4%, respectively. The decrease in strength may be related to the defect in binding the rubber waste to the cement matrix and the increased porosity of the matrix. However, References [47,48,49] show that the use of rubber recyclate in combination with silica fume had a positive effect on the mechanical properties of the concretes obtained.

Although polymeric materials, such as PET, PS, PP, and rubber, were most often used in testing the mechanical properties of concrete, the literature contains several papers on the use of other polymers for this purpose, including PVC [50,51,52,53] or polyurethane [54]. The compressive strength of concrete modified with PVC decreases, regardless of whether it was used in the form of granulate or powder [50,51,52,53]. In addition, the addition of polyurethane reduces the compressive strength of concrete to the control samples. The greatest decrease in strength (86%) was recorded for the series of concrete in which the aggregate was replaced with waste in the amount of 33.7% [54].

In conclusion, it should be stated that, in recent years, there has been a clear increase in the interest of scientists in the use of polymer waste for the production of concrete. This trend is in line with the growing demand for sustainable construction. This is evidenced by review papers [4,5,7] in which the literature discussing the effects of waste plastic aggregates on the strength characteristics of cementitious composites was collected and summarized. The behavior of such composites in aggressive environments, such as freezing/thawing, impact, abrasion, chemical attack, and exposure to elevated temperatures, is discussed. The direction of this research is the result of the systematically increasing amount of polymer waste and the increase in ecological awareness and the need to protect natural resources (replacing aggregate with waste materials). The obtained positive results of research on the use of the post-production waste of thermoplastic elastomers from the production of car mats, presented by us in work [9], allowed us to put forward the thesis that it will also be possible to use elastomer waste from used mats for this purpose, although they slightly differ in chemical composition from waste from the production process. Waste from the production process can be returned to production (waste recirculation), while there is no recycling method for used floor car mats. The use of this waste as a substitute for aggregate without deterioration of the mechanical parameters of concrete seems to be a good direction in the search for methods for the effective reduction of concrete production costs and the use of useless waste.

## 2. Materials and Methods

### 2.1. Materials

**Figure 1:**
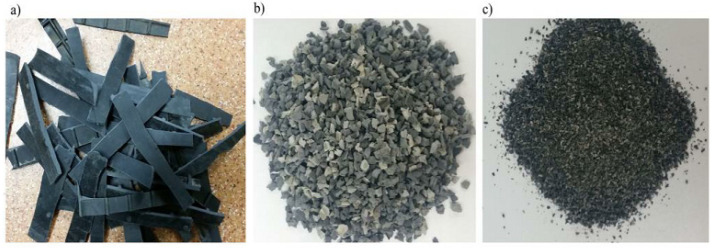
Post-consumer waste from used car liners: preshredded material (**a**); shredded to 2–8 mm fraction (**b**); shredded to 0–2 mm fraction (**c**) [55].

The properties of concrete were modified by the use of post-consumer waste (used car floor mats). Used automobile floor mats were obtained from the Maderpol automobile dismantling plant in Silesian Province, located in Mokra (Poland). After preshredding, the post-consumer waste (Figure 1) was crushed to 0–2 and 2–8 mm fractions in a granulator (SG-2417 SHINI). Both fractions were used in the study. The other materials used for the production of concrete were the same as in our previous work [9], because it is intended to help compare the obtained results. In this research, Portland cement CEM I 42.5R (Cemex) meeting the requirements of PN-EN 197-1, sand of 0–2 mm fraction, gravel aggregate of 2–8 and 8–16 mm fractions, concrete admixtures CHRYSO Plast 331 and CHRYSO Air LB (Company CHRYSO Poland), and water from an intake in Czestochowa (which met the requirements of PN-EN 1008:2004) were used.

### 2.2. Methods

Nine series of concretes were made. An experimental method was used to design the composition of the control concrete (SK) mix. A *c/w* ratio of 2.22 and a consistency class of S2 were assumed. Portland cement CEM I 42.5R, a gravel–sand aggregate mixture with a grain size of 0–16 mm and a sand point of PP = 33.1%, tap water, and plasticizing admixture CHRYSO Plast 331 at 0.35% by weight of cement and aerating admixture CHRYSO Air LB at 0.2% by weight of cement were used for the control concrete. The control concrete (SK) was modified with post-consumer waste thermoplastic elastomer formed from used car floor mats. The material was added in the amount of 2.5, 5.0, 7.5, and 10% by weight of cement, introducing a volumetric adjustment in the weight of sand (series S1–S4) or the weight of gravel aggregate mixture of 2–8 mm (series S5–S8). The concrete series were designated from S1 to S8. The solid components of the concretes were cement (372.5 kg/m^3^), water (167.5 dm^3^/m^3^, plasticizer (1.3 dm^3^/m^3^), and air-entraining admixture (0.745 dm^3^/m^3^), and the content of the variable components is presented in Table 1.

All tests of both concrete mixes (consistency) and hardened concrete (compressive strength, bending strength, tensile strength when splitting, strength loss, and weight loss after frost resistance testing) were carried out per PN-EN standards, described in detail in our previous work [9]. The SEM/EDS analysis of the microstructure of the synthesized concrete composites containing post-consumer waste thermoplastic elastomer was also performed. A scanning electron microscope (SEM) equipped with an X-ray energy-dispersive spectroscopy (EDS)-based chemical composition analysis system was used for the study.

## 3. Results and Discussion

### 3.1. Characteristics of Post-Consumer Waste

**Figure 2:**
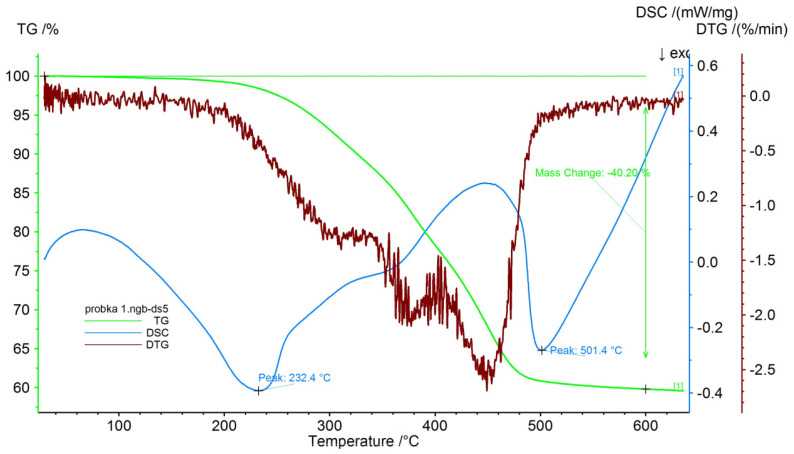
TGA-DTA thermogram of post-consumer thermoplastic elastomer from used car floor mats.

Crushed post-consumer waste of used car mats was used to test the properties of the concretes. Using a WDXRF X-ray spectrometer (Model S8 Tiger from Bruker Company, Billerica, MA, USA), the elemental composition of the thermoplastic elastomer waste used in the study was determined, which is shown in Table 2. The results of the thermogravimetry (TG), differential thermogravimetry (DTG), and differential scanning calorimetry (DSC) of the used waste are shown in Figure 2. The test was carried out in the thermal analysis device Jupiter STA 449 F5 (Netzsch) in a temperature range from 30 °C to 600 °C, with a temperature increase rate of 10 °C/min in an air atmosphere, and with a gas flow rate of 100 cm^3^/min. The curves were analyzed to identify the composition and degradation temperatures of the components in the waste material. The TG curve showed a one-step degradation of the waste elastomer. Two peaks were observed in the DSC curves, a broad peak was seen around 90 °C to 360 °C with a maximum of 232.4 °C and a narrower peak with a maximum of 501.4 °C. The curve clearly shows the initial phase of volatilization of oils, plasticizers, and fillers with a low molecular weight and low boiling point (below 230 °C), as well as a greater weight loss in the temperature range of 230–500 °C, caused by the decomposition of the polymeric material (mostly natural and synthetic styrene–butadiene–rubber). Similar thermogravimetric curves were obtained for commercial rubber products [56,57].

### 3.2. The Consistency of Concrete Mixes

In the first stage of the research, samples of control concrete (SK) were made, assuming a consistency of S2 (cone slump between 50 and 90 mm) and samples of concretes modified with the post-consumer waste of thermoplastic elastomers. After the concrete mixture was prepared, samples were taken to determine the consistency class and air content of the concrete mixture. For the control mix (SK), the cone slump was 70 mm, which classifies it as S2. The air content of this mix was 3.5%. For concrete mixtures modified with the addition of thermoplastic elastomer waste (S1–S8), a cone slump of 50–75 mm was obtained, which also classifies them in the S2 consistency class (Table 3). The air content of the concrete mixtures modified with the tailings was in the range of 3.90–4.6%.

### 3.3. The Mechanical Properties of Concretes

The next stage of the study included the determination of the mechanical strength of concretes modified with waste thermoplastic elastomers. For each series of concrete, 18 cubic specimens of 150 mm sides were made and tested for the compressive strength of the concrete after 7, 28, and 56 days of maturation under laboratory conditions. The standard deviation and 95% confidence interval were determined for the results obtained in each series. The results of compressive strength tests for control and modified concretes are presented in Table 4. After 7 days of concrete curing, a decrease in the compressive strength was observed for all series of waste-modified concretes. Only in the case of concretes containing 2.5% of the waste additive used as a substitute for sand was the hardness comparable to that of the control concrete samples (strength decreased by only 0.64%). The highest decrease in the compressive strength was recorded for concrete samples containing 10% waste, regardless of whether it was used as a sand substitute or as an aggregate substitute, which amounted to 14.16%. A similar tendency of a decrease in the compressive strength was observed after 28 days of maturation of the samples. In the case of concretes containing 2.5% of the waste additive used as a substitute for sand, the compressive strength was comparable to that of the control concrete samples. On the other hand, the decrease in the compressive strength for concrete samples containing 10% of waste used as a sand substitute was 15.09%, and 17.72% for those used as an aggregate substitute. Compressive strength tests after 56 days of maturation of the samples were also carried out for all concrete series. The control concrete (SK) obtained an average compressive strength higher by 8.5% than the 28-day average compressive strength, which was 61.9 ± 1.19 MPa. The concrete of the S1 series, where waste was applied at 2.5%, achieved an average strength compressive strength at the level of the control concrete (62.0 ± 1.9 MPa). However, with the increase in the amount of waste added to the concrete, a decrease in the value of this parameter was observed. The addition of waste to concrete in the amount of 10%, used as a substitute for sand, caused a decrease in the compressive strength by 17.93% and used as a substitute for aggregate with a grain size of 2–8 mm by 12.28%.

Comparing the strength values obtained for concretes (series S5–S8), in which post-consumer waste of thermoplastic elastomers was used as a substitute for aggregate with a grain size of 2–8 mm, we can conclude that they were comparable to the results obtained previously in [9], using post-production waste of these elastomers (waste from the production of car mats). In the case of post-production waste, a decrease in the strength was also observed with an increase in the amount of waste added, ranging from 2.5 to 10%. The compressive strength tested after 7, 28, and 56 days of curing of the modified concrete samples with 2.5% of post-production waste was comparable to the strength of the reference samples (an increase below 1.4%). On the other hand, adding more waste (5.0, 7.5, and 10%) resulted in a decrease in the value of this parameter.

The decrease in the compressive strength of the concrete observed with the increase in the amount of added waste can be explained by the very low bonding force between the surface of waste from thermoplastic elastomers and the cement slurry. Moreover, the hydrophobic nature of polymer waste can inhibit the hydration reaction of cement by limiting the movement (access) of water in the concrete mix. A negative effect on the concrete’s compressive strength parameter was also observed after adding other polymeric materials, such as PET [11,12,14,15,16], EPE [18], EPS [19,20,21,22], and rubber [41,43,45,46,47]. However, some authors report that the addition of PET [10,13], EPS [23,24,25,26,27,28,29,30,31,32,33,34,35,36,37], or PP [32,34,38] wastes increases the compressive strength of concretes modified with this waste.

The addition of waste thermoplastic elastomer to concrete also affects the flexural strength and tensile strength of the concrete (Table 5). The average flexural strength of the S1–S4 series concretes modified with waste thermoplastic elastomer used instead of sand was lower from 5.85 to 6.96% in relation to the control concrete. On the other hand, for the series of concretes S5 and S6 modified with thermoplastic elastomers from post-consumer waste, used as a substitute for 2–8 mm gravel aggregate, in the amount of 2.5 and 5.0% of the cement mass, an increase in the bending strength was observed concerning the series of control concrete, respectively, by 7.24 and 3.64%. While for the series of concretes S7 and S8, in which waste was used in the amount of 7.5 and 10.0%, there was a decrease in this parameter by 4.7 to 5.8%, respectively. The increase in the flexural strength of concrete after adding waste polymers was also noted by other authors. An increase in the bending strength of concretes modified with PET [11,12,14,15] and PP [34,38] was noted. In [13], it was shown that the flexural strength changes markedly with the increase in the amount of added PET waste. The addition of 1% PET increases this parameter by 18.6%, while the addition of 3% decreases it by 19%. On the other hand, the addition of waste LDPE does not affect the flexural strength of concrete [31].

In the concretes of the S1 and S2 concrete series, where post-consumer waste was dosed as a substitute for sand, there was an increase in the average splitting tensile strength. The increase in the average splitting compressive strength was 9.7 and 0.5%. The other series (S3 and S4) showed a decrease in the tested parameter compared to the SK series, by 14.0 and 19.0%, respectively. For the concretes of the S5 and S6 series, where waste was used as a substitute for the 28 mm grain size aggregate in the amount of 2.5 and 5.0%, the average splitting tensile strength was 4.19 ± 0.6 MPa and 3.88 ± 0.1 MPa, respectively. This means that the increase in the splitting strength parameter compared to the control concrete series, for which the strength was 3.78 ± 0.9 MPa, was 10.9 and 2.6%, respectively. For the other series (S7 and S8), there was a decrease in the tested parameter in relation to the SK series, by 4.5% and 5.3%, respectively. As in the case of the compressive strength, both a decrease and an increase in the tensile strength of concrete were observed in the case of the modification with waste polymers [58,59,60]. It has usually been pointed out that these changes depend on the amount of waste used. The declining trend in the tensile strength splitting was usually not as pronounced as for compressive strength.

### 3.4. The Other Tested Properties of Concretes

The next stage of testing was to determine the absorbability, density, pressurized water penetration, frost resistance, and abrasion resistance of the concrete (Table 6). The absorbability test was performed after 28 days of maturation of the samples. For the control concrete (SK), the absorbability was 5.4%. The concretes modified with tailings at 2.5, 5.0, 7.5, and 10% showed similar levels of absorbability (from 4.9 to 5.6%). The concretes modified with post-consumer thermoplastic elastomer wastes have achieved absorption below 9%, which means that these concretes can be used in rooms where they will be protected against direct weather conditions (e.g., floors in halls). Literature reports show that water adsorption in concretes modified with PET increases with the amount of added waste and the size of regranulate [5,17]. However, according to [61], the replacement of sand with aggregate in the amount of 3% by both PET and polycarbonate (PC) does not affect the water absorption or apparent porosity of concrete composites modified with these wastes.

The control concrete (SK), due to the fact of its density, which amounted to 2271 kg/m^3^, was classified as ordinary concrete following PN-EN 206 + A1:2016-12. In addition, all concrete modified with thermoplastic elastomer post-production waste were classified as ordinary concrete, as their density was in the range of 2000–2600 kg/m^3^. The control concrete achieved an average water penetration depth of 65 mm. For the series of concretes modified with waste thermoplastic elastomers, the average depth of the water penetration ranged from 55 mm to 70 mm. The lowest value of the tested parameter (55 mm) was obtained for the S1 series, where 2.5% of post-consumer waste was used, and the highest value (70 mm) was obtained for the S4 series concretes containing 10% of this waste. For the control concrete (SK), the abrasiveness was 7.4 cm^3^/50 cm^2^. For concretes modified with thermoplastic elastomer waste, the abrasion resistance was obtained, ranging from 6.5 to 7.5 cm^3^/50 cm^2^.

**Figure 3:**
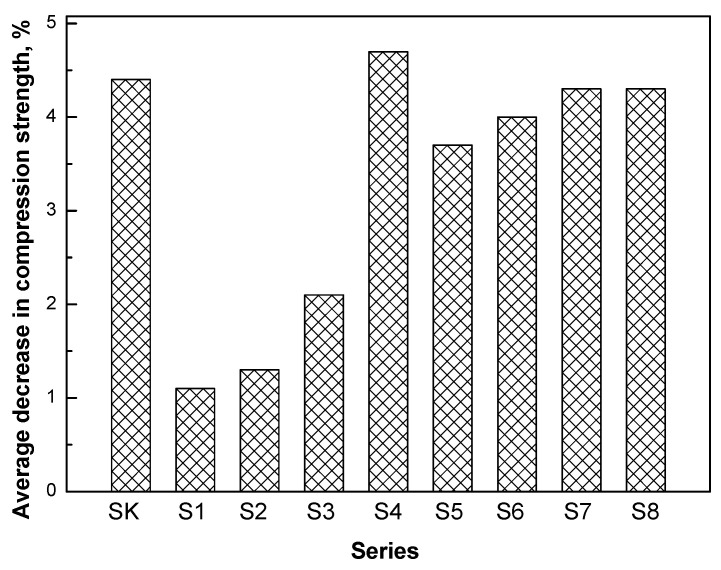
Average decrease in the compressive strength of the concretes after frost resistance testing.

A frost resistance test was conducted for all concretes. For each batch of concrete, 12 cubic specimens of 100 mm sides were made, 6 specimens were subjected to the frost resistance test, and 6 specimens were left in the water as comparison specimens. For the control concrete, the average decrease in the compressive strength after 150 freeze–thaw cycles was 4.4%, while the average weight loss was 0.34% (Figure 3).

The average decrease in the compressive strength for the series of S1–S3 concretes modified with post-consumer waste thermoplastic elastomers used, as a substitute for sand, was lower than the average decrease in the compressive strength of the control concrete. The decrease in the compressive strength for these concretes was 1.1, 1.3, and 2.1%, respectively. In contrast, the average weight loss for these modified series ranged from 0.10% to 0.30% and was lower than the average weight loss for the control concrete (0.34%). For all series of concretes (S5–S8) modified with post-consumer waste car floor mats, used as a substitute for gravel aggregate with a grain size of 2–8 mm, a lower decrease in the compressive strength after frost resistance testing was obtained than for the control concrete. In addition, as shown previously [9], using 2.5 and 5% post-production waste as an aggregate with a grain size of 2–8 mm, a slight decrease in the strength of concrete after frost resistance tests was observed in relation to the control samples (2.4 and 4.3%, respectively), while the addition of 7.5% and 10% waste caused a greater decrease in the compressive strength, by 8.0% and 11.6%, respectively. A lower decrease in the compressive strength after the frost resistance tests in relation to the control samples was also shown by concretes in which the aggregate was partially replaced with the addition of EPS [23], PP [38], and rubber [43].

### 3.5. The Concrete Surface Morphology

**Figure 4:**
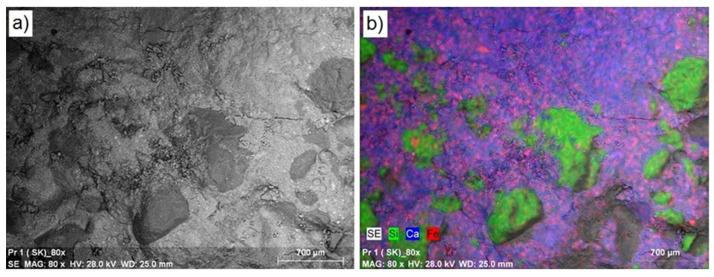
Control concrete microstructure: (**a**) 80 times magnification; (**b**) a map of the location of dominant elements [9].

**Figure 5:**
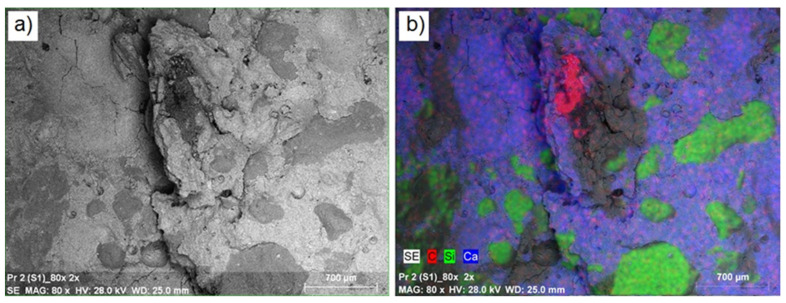
Concrete microstructure S1 series: (**a**) magnification 80 times; (**b**) a map of the location of dominant elements.

In the next stage of the study, the surface layer of the synthesized composites was analyzed, determining mainly their microstructure and elemental composition. Figure 4 shows a microscopic photo of the control concrete (SK) at 80× magnification (Figure 4a), along with maps of the distribution of the dominant elements in this area (Figure 4b). Noticeable in the control concrete (SK) is the lighter structure of the concrete matrix and the darker area representing the silicon-based aggregate. The microstructure at the aggregate–cement matrix interface is compact. As can be seen from the EDS analysis of the concrete surface visible in the photo, in addition to the presence of calcium (33.06%; blue color), a significant content of silicon (20.36%; green color) and iron (4.49%; pink color) can be observed. Aluminum, sulfur, potassium, magnesium, and carbon were present in amounts less than 1.0%. The structures of the concretes modified with post-consumer waste of thermoplastic elastomers from used car floor mats (series S1–S8) did not differ from the structure of the control concrete, as per Figure 5 showing an example structure for the S1 series of concrete. Microscopic images show the compact structure of the cement matrix with both the use of natural aggregate and the use of polymer waste granules. From the EDS analysis, in all the modified concretes, a decrease was observed compared to the control series, in the calcium and silicon content (approximately 4–7%) and an increase in the carbon content (approximately 5% for concrete containing 10% waste), which may be related to the increased amount of post-consumer waste. In the series of concretes where waste thermoplastic elastomers were used, the presence of elements, such as titanium, sodium, and zinc, was also observed, which were not present in the control series. The obtained results are comparable to those previously obtained for concretes with the addition of post-consumer waste of thermoplastic elastomers presented in [9]. Thus, it can be concluded that both post-production and post-consumer wastes of thermoplastic elastomers added in the range of 2.5 to 10% do not significantly affect the change in the microstructure of the tested concretes. In addition, the addition of fly ash from biomass combustion in the amount of 10–30% [62] and waste glass powder in the amount of 25% [63] to concretes does not affect their microstructure. On the other hand, the microstructure of concretes produced with 100% waste glass powder as a substitute for natural sand was characterized by many pores, which resulted from the angular shape of the grain and the smooth surface of the waste sand particles (this resulted in poor bond formation, especially in the interfacial zone). In turn, the microstructure of concretes with waste basalt dust (10–30%) showed a lower porosity in the interfacial transition zone [64].

## 4. Conclusions

The study conducted in this paper on the properties and structure of concrete composites containing post-consumer waste thermoplastic elastomer from used car floor mats and the analysis of the results obtained allows us to conclude that the waste used in the amount of 2.5% can be added as a substitute for sand or as a substitute for gravel aggregate of 2–8 mm grain size for the manufacture of concrete and concrete composites. The addition of waste in this amount does not reduce the parameters of concrete (compressive strength, flexural strength, tensile strength by splitting, frost resistance, and abrasion resistance) with respect to the materials produced without the addition of waste thermoplastic elastomer. The use of waste 0–2 mm fraction in the amount of 2.5% by weight of cement as a substitute for sand allows for reducing the consumption of these aggregates by 20 kg/m^3^, or approximately 5.0%. The microstructure of concretes made based on CEM I 42.5R cement containing the addition of post-consumer waste thermoplastic elastomers in the amount of 2.5% to 10% is characterized by a very similar microstructure to the control concrete made without the addition of waste polymers.

All the waste-modified concretes achieved a lower density than the control concrete, which ranged from 2000 to 2600 kg/m^3^, allowing for the concretes to be classified as ordinary concretes. Both the control concrete and the concretes modified with post-consumer waste thermoplastic elastomer obtained a water absorption rate of approximately 5.0%. Concrete modified with thermoplastic waste obtained from used car mats can be used for elements protected against direct exposure to weather conditions (e.g., industrial floors, tile floors). All concrete modified with post-consumer waste of thermoplastic elastomer met the standard requirements. After performing 150 freeze–thaw cycles, the average decrease in the compressive strength did not exceed 20%, and the average weight loss did not exceed 5%. Thus, it can therefore be concluded that the addition (in the amount of 2.5%) to thermoplastic concrete of an elastomer derived from post-consumer waste, which is currently deposited in landfills, as a substitute for gravel aggregate of 2–8 mm is economically justified and also positively affects the protection of natural resources.

## Figures and Tables

**Table 1:** Composition of the mixture of control concrete and waste-modified concretes as a substitute for sand and gravel aggregate of grain size 2–8 mm.

Composition	Units	Series								
		SK	S1	S2	S3	S4	S5	S6	S7	S8
Sand	kg/m^3^	463.70	443.6	423.6	403.5	384.4	463.7	463.7	463.7	463.7
Gravel 8–16 mm	kg/m^3^	776.20	776.2	776.2	776.2	776.2	776.2	776.2	776.2	776.2
Gravel 2–8 mm	kg/m^3^	635.10	635.1	635.1	635.1	635.1	615.0	594.9	574.9	554.8
Post-consumer waste	kg/m^3^	-	9.31	18.63	27.94	37.25	9.310	18.63	27.94	37.25

**Table 2:** The elemental composition of post-consumer waste, %.

Elemental Composition (% (m/m))											
Ca	Si	Al	Zn	S	Ti	Ba	Fe	K	Sr	C	Other
5.37	1.36	0.93	0.60	0.37	0.09	0.10	0.05	0.06	0.01	78.10	12.92

**Table 3:** Consistency class and air content of the tested concrete mixtures.

Series	Consistence (mm)/Class	Air Content (%)
SK	70/S2	3.50
S1	70/S2	4.30
S2	50/S2	4.20
S3	50/S2	4.15
S4	50/S2	3.90
S5	65/S2	3.90
S6	60/S2	4.20
S7	70/S2	4.60
S8	50/S2	4.00

**Table 4:** Compressive strength of the tested concretes and strength class.

Series	Compression Strength (MPa)			Resistance Class
	After 7 Days	After 28 Days	After 56 Days	
SK	46.6	57.0	61.9	C40/50
S1	46.3	57.0	62.0	C40/50
S2	41.1	50.1	56.2	C35/45
S3	40.7	48.4	51.0	C30/37
S4	40.0	47.4	50.8	C30/37
S5	46.2	57.2	62.1	C40/50
S6	43.9	50.7	56.8	C35/45
S7	42.0	48.5	54.9	C30/37
S8	40.0	46.9	54.3	C30/37

**Table 5:** Flexural tensile strength and splitting tensile strength of concrete.

Series	Flexural Strength (MPa)	Splitting Tensile Strength (MPa)
SK	3.59	3.78
S1	3.38	4.15
S2	3.36	3.8
S3	3.36	3.25
S4	3.34	3.06
S5	3.85	4.19
S6	3.71	3.88
S7	3.42	3.61
S8	3.38	3.58

**Table 6:** Tested parameters for individual concrete series.

Series	Water Absorbability (%)	Density (kg/m^3^)	Water Penetration (mm)	Abrasion Strength (cm^2^/50 cm^2^)
SK	5.4	2271	65	7.4
S1	5.5	2245	55	6.5
S2	5.6	2233	63	6.7
S3	5.5	2220	67	6.9
S4	4.9	2219	70	7.3
S5	5.6	2227	60	6.9
S6	5.5	2222	60	6.9
S7	5.6	2212	65	7.3
S8	5.2	2210	68	7.5

## Data Availability

The data presented in this study are available upon request from the corresponding author.
